# *N*-[4-(Dimethyl­amino)­benzyl­idene]-4*H*-1,2,4-triazol-4-amine

**DOI:** 10.1107/S1600536812014511

**Published:** 2012-04-13

**Authors:** Hui-Liang Zhou, Xiao-Min Zhang

**Affiliations:** a College of Chemistry and Chemical Engineering, Ningxia University, Yinchuan 750021, Ninxia, People’s Republic of China

## Abstract

The title compound, C_11_H_13_N_5_, is a Schiff base synthesized by the reaction of 4-amino-4*H*-1,2,4-triazole and 4-(dimethyl­amino)­benzaldehyde. The dihedral angle between the benzene and triazole rings is 43.09 (11)°. The crystal structure displays weak C—H⋯N inter­actions.

## Related literature

For the biological activity of triazole derivatives, see: Modzelewska & Kalabun (1999 ▶); Rollas *et al.* (1993 ▶); Todoulou *et al.* (1994 ▶); Demirbas *et al.* (2002 ▶); Kahveci *et al.* (2003 ▶). For 4-amino-1,2,4-triazole Schiff bases, see: Desenko & Khim (1995 ▶); Kargin *et al.* (1988 ▶).
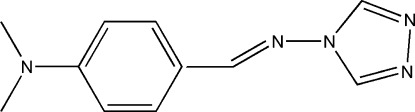


## Experimental

### 

#### Crystal data

C_11_H_13_N_5_
*M* *_r_* = 215.26
Monoclinic, 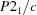

*a* = 10.3665 (16) Å
*b* = 11.1585 (19) Å
*c* = 9.5248 (12) Å
β = 90.257 (1)°
*V* = 1101.8 (3) Å^3^
*Z* = 4
Mo *K*α radiation
μ = 0.08 mm^−1^
*T* = 298 K
0.52 × 0.15 × 0.11 mm

#### Data collection

Bruker SMART CCD area-detector diffractometer
Absorption correction: multi-scan (*SADABS*; Bruker, 2002 ▶) *T*_min_ = 0.957, *T*_max_ = 0.991
5465 measured reflections
1940 independent reflections
1184 reflections with *I* > 2σ(*I*)
*R*_int_ = 0.062

#### Refinement

*R*[*F* ^2^ > 2σ(*F* ^2^)] = 0.047
*wR*(*F* ^2^) = 0.119
*S* = 1.00
1940 reflections
148 parameters
H-atom parameters constrained
Δρ_max_ = 0.17 e Å^−3^
Δρ_min_ = −0.18 e Å^−3^

### 

Data collection: *SMART* (Bruker, 2002 ▶); cell refinement: *SAINT* (Bruker, 2002 ▶); data reduction: *SAINT*; program(s) used to solve structure: *SHELXS97* (Sheldrick, 2008 ▶); program(s) used to refine structure: *SHELXL97* (Sheldrick, 2008 ▶); molecular graphics: *SHELXTL* (Sheldrick, 2008 ▶); software used to prepare material for publication: *SHELXTL*.

## Supplementary Material

Crystal structure: contains datablock(s) I, global. DOI: 10.1107/S1600536812014511/ff2062sup1.cif

Structure factors: contains datablock(s) I. DOI: 10.1107/S1600536812014511/ff2062Isup2.hkl

Supplementary material file. DOI: 10.1107/S1600536812014511/ff2062Isup3.cml

Additional supplementary materials:  crystallographic information; 3D view; checkCIF report

## Figures and Tables

**Table 1:** Hydrogen-bond geometry (Å, °)

*D*—H⋯*A*	*D*—H	H⋯*A*	*D*⋯*A*	*D*—H⋯*A*
C1—H1⋯N4^i^	0.93	2.57	3.448 (3)	157
C2—H2⋯N2^ii^	0.93	2.43	3.284 (3)	152
C11—H11*B*⋯N1^iii^	0.96	2.60	3.543 (3)	166

Symmetry codes: (i) 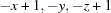; (ii) 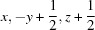; (iii) 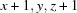.

**Fig. 1.**
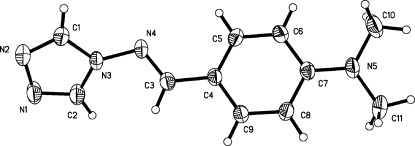
The molecular structure of the title compound, showing 30% displacement ellipsoids for the non-hydrogen atoms. Hydrogen atoms are drawn as spheres of arbitrary radius.

## supplementary crystallographic information

### Comment

1,2,4-Triazole and their derivatives have been used as starting materials for synthesis of many heterocycles. The aroyl Schiff bases of 4-amino-1,2,4-triazole have received considerable attention over the past few decades (Desenko *et al.*, 1995; Kargin *et al.*, 1988; Modzelewska & Kalabun, 1999). In recent years, various 1,2,4-triazoles and their derivatives have been found to be associated with diverse pharmacological activities such as anticonvulsant, antifungal, anticancer, anti-inflammatory and antibacterial (Rollas *et al.*, 1993; Todoulou *et al.*, 1994). The present X-ray crystal structure analysis was undertaken in order to study the stereochemistry and crystal packing of the title compound (I).

The molecular structure and the atom-numbering scheme of the title compound are shown in Fig. 1. In the molecule, all bond lengths and angles are normal. As shown in Fig 1, the title compound is composed of two planar segments. One segment is a triazole ring, which is contains N3, C1, N2, C2, N1, and another segment is a benzene ring. The dihedral angle between the two planar segments is 43.09 (11)°. In the triazole ring, the N1=C2 and N2=C1 bonds display double-bond character, with bond distances of 1.303 (3) and 1.295 (2) Å, respectively.

### Experimental

A mixture of 4-amino-4*H*-1,2,4-triazole 1 (0.51 g, 6 mmol) and 4-Dimethylaminobenzaldehyde (0.85 g, 6 mmol) was reacted in 40 ml ethanol at 353 K for 0.3 h. Single crystals suitable for X-ray diffraction analysis were obtained by slow evaporation of the ethanol solution.

### Refinement

The H atoms were positioned geometrically, with C—H distances of 0.93–0.96 Å for aromatic, methylene and methyl H atoms, respectively, and *U*_iso_(H) = 1.2–1.5*U*_eq_ of the parent atom.

### Crystal data

**Table:**

C_11_H_13_N_5_	*F*(000) = 456
*M**_r_* = 215.26	*D*_x_ = 1.298 Mg m^−^^3^
Monoclinic, *P*2_1_/*c*	Mo *K*α radiation, λ = 0.71073 Å
Hall symbol: -P 2ybc	Cell parameters from 1214 reflections
*a* = 10.3665 (16) Å	θ = 2.7–23.0°
*b* = 11.1585 (19) Å	µ = 0.08 mm^−^^1^
*c* = 9.5248 (12) Å	*T* = 298 K
β = 90.257 (1)°	Cuboid, colourless
*V* = 1101.8 (3) Å^3^	0.52 × 0.15 × 0.11 mm
*Z* = 4	

### Data collection

**Table:**

Bruker SMART CCD area-detector diffractometer	1940 independent reflections
Radiation source: fine-focus sealed tube	1184 reflections with *I* > 2σ(*I*)
Graphite monochromator	*R*_int_ = 0.062
phi and ω scans	θ_max_ = 25.0°, θ_min_ = 2.0°
Absorption correction: multi-scan (*SADABS*; Bruker, 2002)	*h* = −12→12
*T*_min_ = 0.957, *T*_max_ = 0.991	*k* = −13→9
5465 measured reflections	*l* = −11→11

### Refinement

**Table:**

Refinement on *F*^2^	Secondary atom site location: difference Fourier map
Least-squares matrix: full	Hydrogen site location: inferred from neighbouring sites
*R*[*F*^2^ > 2σ(*F*^2^)] = 0.047	H-atom parameters constrained
*wR*(*F*^2^) = 0.119	*w* = 1/[σ^2^(*F*_o_^2^) + (0.0462*P*)^2^] where *P* = (*F*_o_^2^ + 2*F*_c_^2^)/3
*S* = 1.00	(Δ/σ)_max_ < 0.001
1940 reflections	Δρ_max_ = 0.17 e Å^−^^3^
148 parameters	Δρ_min_ = −0.18 e Å^−^^3^
0 restraints	Extinction correction: *SHELXL97* (Sheldrick, 2008), Fc^*^=kFc[1+0.001xFc^2^λ^3^/sin(2θ)]^-1/4^
Primary atom site location: structure-invariant direct methods	Extinction coefficient: 0.086 (5)

### Special details

**Table:**

Geometry. All e.s.d.'s (except the e.s.d. in the dihedral angle between two l.s. planes) are estimated using the full covariance matrix. The cell e.s.d.'s are taken into account individually in the estimation of e.s.d.'s in distances, angles and torsion angles; correlations between e.s.d.'s in cell parameters are only used when they are defined by crystal symmetry. An approximate (isotropic) treatment of cell e.s.d.'s is used for estimating e.s.d.'s involving l.s. planes.
Refinement. Refinement of *F*^2^ against ALL reflections. The weighted *R*-factor *wR* and goodness of fit *S* are based on *F*^2^, conventional *R*-factors *R* are based on *F*, with *F* set to zero for negative *F*^2^. The threshold expression of *F*^2^ > σ(*F*^2^) is used only for calculating *R*-factors(gt) *etc*. and is not relevant to the choice of reflections for refinement. *R*-factors based on *F*^2^ are statistically about twice as large as those based on *F*, and *R*- factors based on ALL data will be even larger.

### Fractional atomic coordinates and isotropic or equivalent isotropic displacement parameters (Å^2^)

**Table:**

	*x*	*y*	*z*	*U*_iso_*/*U*_eq_	
N1	0.20167 (17)	0.23656 (19)	0.4358 (2)	0.0665 (6)	
N2	0.24339 (17)	0.13578 (17)	0.36475 (19)	0.0599 (5)	
N3	0.34969 (14)	0.14765 (14)	0.56204 (17)	0.0461 (4)	
N4	0.43764 (14)	0.11434 (15)	0.66817 (17)	0.0498 (5)	
N5	0.87803 (15)	0.16192 (15)	1.14369 (18)	0.0590 (5)	
C1	0.33047 (19)	0.08539 (19)	0.4428 (2)	0.0540 (6)	
H1	0.3740	0.0154	0.4195	0.065*	
C2	0.26690 (19)	0.2406 (2)	0.5530 (2)	0.0588 (6)	
H2	0.2576	0.2996	0.6212	0.071*	
C3	0.49452 (17)	0.20274 (18)	0.7270 (2)	0.0469 (5)	
H3	0.4751	0.2797	0.6959	0.056*	
C4	0.58726 (17)	0.18934 (17)	0.8392 (2)	0.0437 (5)	
C5	0.61841 (19)	0.07978 (19)	0.8997 (2)	0.0539 (6)	
H5	0.5744	0.0114	0.8707	0.065*	
C6	0.7122 (2)	0.06956 (19)	1.0008 (2)	0.0581 (6)	
H6	0.7301	−0.0051	1.0396	0.070*	
C7	0.78200 (17)	0.17091 (18)	1.0468 (2)	0.0458 (5)	
C8	0.74731 (17)	0.28123 (18)	0.9884 (2)	0.0491 (6)	
H8	0.7890	0.3504	1.0186	0.059*	
C9	0.65320 (18)	0.28964 (18)	0.8876 (2)	0.0477 (6)	
H9	0.6329	0.3644	0.8505	0.057*	
C10	0.9193 (3)	0.0473 (2)	1.1988 (3)	0.0853 (9)	
H10A	0.8525	0.0144	1.2566	0.128*	
H10B	0.9963	0.0579	1.2539	0.128*	
H10C	0.9366	−0.0064	1.1224	0.128*	
C11	0.9429 (2)	0.2687 (2)	1.1945 (2)	0.0691 (7)	
H11A	0.9861	0.3074	1.1180	0.104*	
H11B	1.0049	0.2468	1.2652	0.104*	
H11C	0.8806	0.3225	1.2340	0.104*	

### Atomic displacement parameters (Å^2^)

**Table:**

	*U*^11^	*U*^22^	*U*^33^	*U*^12^	*U*^13^	*U*^23^
N1	0.0658 (12)	0.0829 (14)	0.0507 (13)	0.0148 (10)	−0.0210 (10)	−0.0039 (11)
N2	0.0641 (11)	0.0706 (13)	0.0448 (12)	−0.0003 (10)	−0.0169 (9)	0.0003 (10)
N3	0.0464 (9)	0.0559 (11)	0.0359 (10)	−0.0001 (8)	−0.0119 (8)	0.0019 (9)
N4	0.0521 (10)	0.0569 (11)	0.0403 (11)	0.0002 (8)	−0.0165 (8)	0.0024 (9)
N5	0.0578 (10)	0.0678 (13)	0.0512 (12)	0.0076 (10)	−0.0251 (9)	−0.0019 (10)
C1	0.0651 (13)	0.0536 (13)	0.0432 (14)	−0.0038 (11)	−0.0132 (11)	−0.0005 (11)
C2	0.0547 (13)	0.0712 (16)	0.0503 (15)	0.0103 (12)	−0.0140 (11)	−0.0079 (12)
C3	0.0441 (11)	0.0530 (13)	0.0436 (14)	0.0054 (10)	−0.0059 (10)	−0.0006 (11)
C4	0.0454 (11)	0.0481 (12)	0.0374 (13)	0.0048 (9)	−0.0075 (9)	−0.0018 (10)
C5	0.0642 (13)	0.0516 (13)	0.0456 (14)	−0.0066 (10)	−0.0164 (11)	−0.0038 (11)
C6	0.0744 (14)	0.0508 (13)	0.0488 (14)	0.0075 (11)	−0.0204 (12)	0.0025 (11)
C7	0.0472 (11)	0.0536 (13)	0.0364 (13)	0.0077 (10)	−0.0068 (9)	−0.0039 (10)
C8	0.0462 (11)	0.0535 (13)	0.0475 (14)	−0.0016 (10)	−0.0104 (10)	−0.0063 (11)
C9	0.0489 (11)	0.0493 (13)	0.0448 (14)	0.0051 (9)	−0.0099 (10)	−0.0002 (10)
C10	0.0898 (17)	0.089 (2)	0.0766 (19)	0.0287 (15)	−0.0392 (15)	0.0001 (16)
C11	0.0548 (13)	0.0922 (18)	0.0602 (17)	−0.0066 (12)	−0.0212 (12)	−0.0041 (14)

### Geometric parameters (Å, º)

**Table:**

N1—C2	1.303 (3)	C4—C9	1.389 (3)
N1—N2	1.383 (2)	C5—C6	1.370 (3)
N2—C1	1.295 (2)	C5—H5	0.9300
N3—C1	1.346 (2)	C6—C7	1.411 (3)
N3—C2	1.349 (2)	C6—H6	0.9300
N3—N4	1.408 (2)	C7—C8	1.397 (3)
N4—C3	1.277 (2)	C8—C9	1.369 (3)
N5—C7	1.358 (2)	C8—H8	0.9300
N5—C10	1.446 (3)	C9—H9	0.9300
N5—C11	1.450 (3)	C10—H10A	0.9600
C1—H1	0.9300	C10—H10B	0.9600
C2—H2	0.9300	C10—H10C	0.9600
C3—C4	1.442 (3)	C11—H11A	0.9600
C3—H3	0.9300	C11—H11B	0.9600
C4—C5	1.389 (3)	C11—H11C	0.9600
			
C2—N1—N2	106.54 (17)	C5—C6—C7	120.83 (19)
C1—N2—N1	106.92 (17)	C5—C6—H6	119.6
C1—N3—C2	104.55 (17)	C7—C6—H6	119.6
C1—N3—N4	124.23 (17)	N5—C7—C8	121.48 (18)
C2—N3—N4	131.21 (17)	N5—C7—C6	121.64 (18)
C3—N4—N3	114.03 (17)	C8—C7—C6	116.88 (18)
C7—N5—C10	121.78 (18)	C9—C8—C7	121.39 (19)
C7—N5—C11	120.24 (17)	C9—C8—H8	119.3
C10—N5—C11	117.98 (18)	C7—C8—H8	119.3
N2—C1—N3	111.1 (2)	C8—C9—C4	121.70 (19)
N2—C1—H1	124.4	C8—C9—H9	119.1
N3—C1—H1	124.4	C4—C9—H9	119.1
N1—C2—N3	110.9 (2)	N5—C10—H10A	109.5
N1—C2—H2	124.6	N5—C10—H10B	109.5
N3—C2—H2	124.6	H10A—C10—H10B	109.5
N4—C3—C4	123.37 (19)	N5—C10—H10C	109.5
N4—C3—H3	118.3	H10A—C10—H10C	109.5
C4—C3—H3	118.3	H10B—C10—H10C	109.5
C5—C4—C9	117.30 (18)	N5—C11—H11A	109.5
C5—C4—C3	123.47 (18)	N5—C11—H11B	109.5
C9—C4—C3	119.19 (18)	H11A—C11—H11B	109.5
C6—C5—C4	121.84 (19)	N5—C11—H11C	109.5
C6—C5—H5	119.1	H11A—C11—H11C	109.5
C4—C5—H5	119.1	H11B—C11—H11C	109.5
			
C2—N1—N2—C1	0.1 (2)	C3—C4—C5—C6	176.31 (19)
C1—N3—N4—C3	143.42 (19)	C4—C5—C6—C7	−0.5 (3)
C2—N3—N4—C3	−38.0 (3)	C10—N5—C7—C8	−176.5 (2)
N1—N2—C1—N3	−0.3 (2)	C11—N5—C7—C8	3.2 (3)
C2—N3—C1—N2	0.5 (2)	C10—N5—C7—C6	3.8 (3)
N4—N3—C1—N2	179.38 (16)	C11—N5—C7—C6	−176.49 (19)
N2—N1—C2—N3	0.2 (2)	C5—C6—C7—N5	−177.92 (19)
C1—N3—C2—N1	−0.4 (2)	C5—C6—C7—C8	2.4 (3)
N4—N3—C2—N1	−179.24 (17)	N5—C7—C8—C9	177.99 (18)
N3—N4—C3—C4	179.31 (16)	C6—C7—C8—C9	−2.3 (3)
N4—C3—C4—C5	−3.8 (3)	C7—C8—C9—C4	0.4 (3)
N4—C3—C4—C9	173.89 (18)	C5—C4—C9—C8	1.5 (3)
C9—C4—C5—C6	−1.4 (3)	C3—C4—C9—C8	−176.33 (18)

### Hydrogen-bond geometry (Å, º)

**Table:**

*D*—H···*A*	*D*—H	H···*A*	*D*···*A*	*D*—H···*A*
C1—H1···N4^i^	0.93	2.57	3.448 (3)	157
C2—H2···N2^ii^	0.93	2.43	3.284 (3)	152
C11—H11*B*···N1^iii^	0.96	2.60	3.543 (3)	166

Symmetry codes: (i) −*x*+1, −*y*, −*z*+1; (ii) *x*, −*y*+1/2, *z*+1/2; (iii) *x*+1, *y*, *z*+1.
